# Corneal perforation: the most feared complication of corneal abscess

**DOI:** 10.11604/pamj.2020.35.34.14754

**Published:** 2020-02-07

**Authors:** Ihsane Sabrane, Lalla Oufae Cherkaoui

**Affiliations:** 1 Université Mohammed V Souissi, Service d’Ophtalmologie A de l’Hôpital des Spécialités, Centre Hospitalier Universitaire, Rabat, Maroc

**Keywords:** Cornea, perforation, hernia

## Image in medicine

We report the case of a 60-year-old patient who consults for eye pain after 3-months of trauma to the right eye. The examination finds a visual acuity with light perception, a perforated cornea in the center with hernia of the iris and a corneal halos abscessed all around (A, B). Corneal abscesses are a serious pathology and potentially blinding, their risk factors are multiple: contact lens wear, corneal trauma, dry eye with blepharitis, corneal anesthesia and keratoplasties. The severity factors requiring hospitalization with corneal harvesting and treatment with fortified antibiotic eye drops adapted to the antibiogram are: an abscess 3mm or more long axis, a central location or less than 2mm from the center, an inflammatory reaction of the anterior chamber. Corneal abscesses appearing at a distance from trauma, as the case we describe are often of fungal origin and are generally of very poor prognosis.

**Figure 1:**
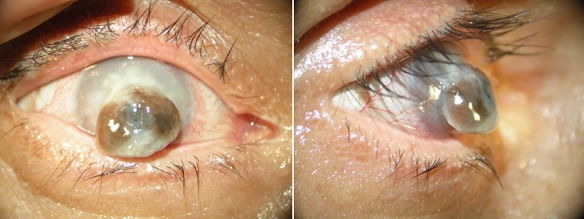
Corneal perforation with hernia of the iris: A) front view; B) profile view.

